# Once I get on a puzzle, I can't get off: Cachexia and wasting in 2018

**DOI:** 10.1002/jcsm.12366

**Published:** 2018-11-18

**Authors:** Stephan von Haehling, Stefan D. Anker

**Affiliations:** ^1^ Department of Cardiology and Pneumology University of Göttingen Medical School Göttingen Germany; ^2^ Department of Cardiology (CVK); and Berlin‐Brandenburg Center for Regenerative Therapies (BCRT); German Centre for Cardiovascular Research (DZHK) partner site Berlin Charité Universitätsmedizin Berlin Germany

Cachexia and wasting disorders can still be considered a niche in the medical arena. The Cachexia Conference, held this year in Maastricht, the Netherlands, from December 7 to 9, has helped establishing this field in clinical and basic research. The conference was founded in 2000 with some 100 attendees in Berlin, Germany. The second conference took place in Berlin again with the number of participants already doubled. Later venues included Rome, Italy, in 2005 and Tampa, Florida, in 2007. The initial Cachexia Conferences were held every other year with the number of attendees increasing to some 426 in Paris, France, in 2015. Of course, the location of the conference does play a major role in the decision to participate or not, and therefore, it is not a surprise that the Kobe Conference in 2013 had a slightly lower number of participants than previous ones. The increasing interest in the field of wasting, however, is also highlighted by the increasing number of abstracts presented at the Cachexia Conference from 197 at the meeting in Milan in 2011 to 250 at the meeting in Rome last year. Of note, these abstracts presented in Rome came from no less than 37 different countries around the globe.

Of course, it has been hard work to reach the point where the conference stands right now. Establishing a field such as cachexia cannot be done by a conference alone, even when it becomes annual like the Cachexia Conference did in 2015. The conference can and should be viewed as only one piece in the huge cachexia puzzle. Or, as the British coiffeur Vidal Sassoon (1928–2012) once said: ‘The only place where success comes before work is in the dictionary’.

Another piece in the cachexia puzzle is the not‐for‐profit *Society on Cachexia and Wasting Disorders* (SCWD) that was founded in November 2008. Its existence underscores the strong determination of the founders of the SCWD to carry out their mission: (i) increase public awareness of cachexia and wasting disease, (ii) educate health care professionals and the public about cachexia and wasting disease, (iii) promote research and the development of new treatments for cachexia and (iv) provide an international forum for the interdisciplinary academic exchange of new ideas and information. With its 150 members at its 10th anniversary, the society is determined to fulfil its duty in achieving these aims. The American physicist and Nobel Prize winner Richard P. Feynman (1918–1988) said ‘Once I get on a puzzle, I can't get off’.^1^ In fact, with treatments barely established, we are not even in the middle of piecing together the cachexia puzzle. The *Journal of Cachexia, Sarcopenia and Muscle* (JCSM) was launched in September 2010, which means that we are publishing in the 9th year. A total of 451 entries can be found for the journal in PubMed, and their number is steadily increasing.^2^ With an impact factor of 12.511, the interest in the broad field of cachexia and wasting is strongly underscored. Two daughter journals have been founded as well: JCSM—Clinical Reports (http://www.jcsm-clinical-reports.info) started in July 2016 and is dedicated to clinical reports in the strictest sense of the word, that is, original and review papers from the clinical field of wasting disorders in the broadest sense including case reports.^3^ The other one, JCSM—Rapid Communications (http://www.jcsm-rapid-communications.info), was launched a little later in January this year and is supposed to publish scientific papers from a very broad field including original and review papers from clinical as well as basic science groups.

Of course, we cannot claim to hold all the pieces to the cachexia puzzle. Researchers from around the globe must work together in tackling ‘the last illness’, as Corie Lok recently named cachexia^4^ so that mortality and morbidity can be improved. If the Cachexia Conference can be the podium for such undertaking, we would be truly delighted. In this sense and having quoted a hairdresser as well as a Nobel Prize winner:

Welcome to the 11th Cachexia Conference!

## Conflict of interest

None declared.
